# Therapist Delivery of Evidence Based Practice (EBP) Strategies in a System-Driven Implementation of Multiple EBPs for Children

**DOI:** 10.1007/s10488-026-01501-1

**Published:** 2026-04-27

**Authors:** Teresa Lind, Anna Lau, Julia Cox, Colby Chlebowski, Joyce Lui, Y. Vivian Byeon, Lauren Brookman-Frazee

**Affiliations:** 1 San Diego State University, San Diego, USA; 2 Child and Adolescent Services Research Center, San Diego, USA; 3 University of California, Los Angeles, Los Angeles, USA; 4 Concordia University, Montreal, Canada; 5 University of California, San Diego, San Diego, USA

**Keywords:** Evidence-based practice, Community mental health, Therapist self-efficacy

## Abstract

Examples of system-driven implementation of multiple evidence-based practices (EBPs) are becoming more frequent in efforts to improve community mental health care, but there is limited understanding of how these efforts change community therapist practice. Within one such implementation effort in the Los Angeles County Department of Mental Health, this observational study sought to: (1) examine observer ratings of therapist delivery of EBP strategies in sessions where therapists claimed reimbursement to one of six youth mental health EBPs; and (2) explore factors associated with extensiveness of EBP strategy delivery. Data were drawn from 680 sessions with 273 youth delivered by 103 therapists in 14 agencies. Sessions were audio recorded and rated by trained observers for extensiveness of therapist delivery of EBP technique and content strategies. Therapists were observed to use EBP techniques (Structuring Treatment Techniques and Skill Building Techniques) in the majority of sessions. Consistent with expectations, use of specific EBP content strategies were more targeted and less commonly observed at every session. Finally, the following factors were associated with higher observed EBP strategy extensiveness: sessions completed in a non-English language (compared with sessions completed in English), sessions using an EBP with a prescribed session content/order (compared with sessions using an EBP without a prescribed session content/order), higher levels of therapist reported self-efficacy with the EBP being delivered, older client age, and therapists with a cognitive behavioral theoretical orientation (compared with therapists with an orientation other than cognitive behavioral). Patterns of therapist EBP strategy delivery followed expected patterns, with techniques strategies used in the majority of sessions and specific content strategies used in a more targeted fashion. Given the dearth of observational measurement of EBP implementation-as-usual, these findings contribute to our understanding of key outcomes of scale-up efforts. Predictors of EBP strategy use have implications for targeting efforts to promote sustainment, such as improving provider EBP self-efficacy.

In recent years, efforts to improve mental health services in community settings have employed large-scale implementation efforts of evidence-based practices (EBPs) (Hoagwood et al., 2014). Typically these initiatives involve training community therapists in multiple EBPs to ensure that therapists can address the range of needs of clients presenting for treatment (Chorpita et al., 2011; Lau & Brookman-Frazee, 2016; Nakamura et al., 2014; Powell et al., 2016). However, there is significant heterogeneity in the pre-service education, in-service training, and practice of community mental health clinicians, and there is limited available data regarding how therapists deliver EBP strategies in session after initial training (Becker-Haimes et al., 2019). This information can provide insight about the impact and sustainment of scale-up efforts and help quantify implementation outcomes in youth community mental health (Lau & Brookman-Frazee, 2016).

## Observational Coding of Therapist Strategy Use

Observational coding of therapy sessions is considered the gold standard method to assess therapist delivery of strategies, including the constructs of treatment fidelity and adherence (Schoenwald & Garland, 2013). This method involves training a team of independent observers to review session recordings and code a defined set of intervention strategies while demonstrating inter-observer reliability. Although observation may provide valuable information about EBP implementation, this method is time and labor-intensive and rarely used outside controlled trial contexts (Baldwin et al., 2013; Imel et al., 2014). Due to these challenges, opportunities to assess therapist EBP strategy delivery in community service systems using this method are rare.

One notable exception is a project aimed at developing treatment integrity measures of cognitive behavioral therapy (CBT) for youth anxiety using therapy recordings from community mental health clinics and controlled trial research settings (McLeod et al., 2015; Southam-Gerow et al., 2016). Part of this project compared observed delivery of EBP strategies across the community and research settings and found that community clinicians’ delivery of EBP strategies waned over the course of their cases relative to clinicians in the research setting (Smith et al., 2017). Observational coding also revealed that community-based clinicians delivered EBP strategies at a lower level of extensiveness compared with research-setting-based clinicians (Southam-Gerow et al., 2010, 2016). This project highlighted the importance of both context and training for the observed delivery of a specific EBP such as CBT for youth anxiety (Southam-Gerow et al., 2010). Capturing community EBP implementation-as-usual following *system*-driven dissemination and training efforts is even more limited, and of considerable importance given the major cost of these investments in public systems of care.

## Coding Therapist Use of Essential EBP Strategies

Given the resource burden involved in the observational measurement of even one EBP, there is increased challenge in attempting to measure EBP delivery in a multiple-EBP implementation context. Individual EBPs typically feature an array of strategies to deliver therapeutic content, often in a particular sequence, to address a specific mental health target problem. To measure the delivery of a single EBP requires tremendous resources to develop, train, and sustain reliable observational coding efforts. An alternative approach is to focus on measuring EBP strategies that are present in multiple EBPs designed to address specific treatment targets. This is consistent with the “common elements” approach to identifying treatment components contained across many effective interventions for a given mental health target (Boustani et al. 2017; Chorpita and Daleiden 2009; Garland et al. 2008a, b). Common elements are distinct clinical strategies, such as cognitive restructuring or exposure, that are frequently included in EBPs for a given problem focus. Observational coding systems focused on cross-cutting strategies have been used to characterize therapist strategies in both usual care (Garland et al. 2010a, b) and in EBP implementation efforts (McLeod et al., 2015; McLeod & Weisz, 2005). The observational coding strategy used in the current study is based on these prior methods (Brookman-Frazee et al., 2021).

### Coding EBP Strategies: Content and Technique

Many coding systems designed to measure therapist delivery of EBP strategies across multiple EBPs make a key distinction between strategies related to content and technique (Brookman-Frazee et al. 2021; McLeod et al. 2018a, b). Content refers to the substance or issue being addressed in the therapeutic intervention, and often consists of new knowledge, skills, and experiences (McLeod et al. 2018a, b; Morgan et al. 2018). Content strategies refer to *what* is being taught in therapy. Examples include cognitive restructuring, problem-solving steps, and praise. In general, any given content strategy is not expected to be covered in every session (Kolko et al., 2011; Morgan et al., 2018). Rather, a therapist may be expected to spend one or two sessions focusing on a content strategy in-depth, and then review and consolidate in selected later sessions.

Technique strategies refer to *how* material is taught in therapy, or the active ways a therapist attempts to impart content, or relate to, a client (Garland et al. 2010a, b). Examples include modeling, role play, and in vivo coaching. Techniques are expected to be used across most treatment sessions, as these strategies are integral to effectively teach the treatment content (Garland et al., 2010a; Southam-Gerow et al., 2016). For example, if a therapist is teaching a caregiver the treatment content of increasing praise, the therapist might use techniques including *modeling* how to praise a youth, participate in a *role play* pretending to be the youth so that the parent can practice praise, and assigning *homework* to practice praise outside of session. Recent work has described observed differences in both quantity and quality of different passive and active technique strategies for an individual EBP over the course of treatment and by setting, with lower levels of active technique strategies in community settings compared with research settings (Cox et al., 2020). Presumably, both EBP content and technique strategies can influence the effectiveness of an intervention, and it is therefore critical to assess both and understand factors that may influence their use (Garland et al. 2010a, b; McLeod et al. 2018a, b; Southam-Gerow et al. 2016).

## Conceptual Framework of EBP Implementation

One model which can aid in conceptualizing factors that may impact therapist delivery of EBP strategies is the Exploration, Preparation, Implementation, Sustainment (EPIS) framework, which theorizes that the fit of the EBP within the system (outer context), as well as the fit within the organization (inner context), shape EBP implementation (Aarons et al., 2011; Moullin et al., 2019). EPIS focuses on influences at multiple levels that can impact EBP implementation, including session/EBP-, client-, therapist-, and organization-level factors at different phases of implementation (Aarons et al., 2011). Guided by EPIS, the current study examined how variation in inner context factors, including session/EBP-, client-, and therapist-level factors are associated with observed therapist delivery of EBP strategies during the sustainment phase of a large-scale implementation effort.

## Multilevel Associations with Observed Therapist EBP Strategy Delivery

### Session/EBP-Level Factors Associated with Observed Therapist EBP Strategy Delivery

One EBP-level factor that varies within community implementation efforts is the extent to which providers receive ongoing support and consultation following an initial EBP training workshop. There is some evidence that provision and receipt of ongoing consultation is linked with higher levels of therapist EBP strategy delivery. For example, ongoing consultation has been associated with higher observed therapist use of technique strategies such as in vivo coaching following training in Attachment and Biobehavioral Catch-up (ABC; Caron & Dozier, 2019). Multiple studies have found higher overall observed fidelity scores for therapists who participate in ongoing consultation compared to an initial workshop only in parent training interventions (Webster-Stratton et al., 2014) and Cognitive Behavioral Therapy (CBT; Beidas et al., 2014; Edmunds et al., 2017; Sholomskas et al., 2008). However, there have been some notable exceptions. Ongoing supervision for Motivational Interviewing was associated with therapist-reported but not observer-rated EBP strategy delivery (Wain et al., 2015) and therapist consultation attendance and receipt of specific consultation activities were not associated with therapist fidelity to Cognitive Processing Therapy (CPT; Mallard Swanson et al., 2021).

### Client-Level Factors Associated with Observed Therapist EBP Strategy Delivery

Given the diversity and complexity of mental health presentations of youth served in public mental health systems, it may be important to identify client-level factors, such as race/ethnicity, language, developmental level, or problem focus, that may impact therapist EBP implementation. However, the literature regarding the relationship between client-level factors and observed therapist EBP strategy delivery is sparse, and lags behind the likewise limited study of client factors associated with EBP effectiveness (Callahan et al., 2013). Using caregivers’ ratings of therapist strategy use, studies of Multisystemic Therapy (MST) have found that therapist-youth race/ethnicity match was associated with higher levels of therapist EBP strategy delivery (Chapman & Schoenwald, 2011; Schoenwald et al., 2003), but there were no associations with youth age (Schoenwald et al., 2003, 2005). A study of Pivotal Response Treatment for children with autism found no relation between youth characteristics (age and symptom severity) and observed therapist EBP fidelity (Verschuur et al., 2020), despite previous studies suggesting otherwise (Irvin et al., 2015; Suhrheinrich et al., 2016). Identifying client-level predictors of EBP strategy delivery may point attention to where implementation support is indicated.

### Therapist-Level Factors Associated with Observed Therapist EBP Strategy Delivery

There has been more research examining the association between therapist-level factors and observed EBP delivery. Therapist education and clinical training background have been found to be associated with observed therapist EBP strategy delivery. In a community implementation of Twelve Step Facilitation, therapists with graduate degrees and therapists who reported higher levels of self-efficacy with counseling skills had significantly higher observer EBP strategy delivery compared with therapists without graduate degrees and those with lower counseling self-efficacy (Campbell et al., 2013). Similarly, postgraduate training, national certification, and more years of clinical experience were associated with greater use of observed therapist EBP strategy delivery in a community implementation of CBT (Ginsburg et al., 2019). However, therapist experience has not been consistently associated with implementation outcomes. In fact, years of experience was related to lower observed CBT adherence in a community mental health sample in Philadelphia (Beidas et al., 2014). Length of clinical experience may obscure specific factors that impact fidelity, such as type of previous experience, skills emphasized in training, and exposure to EBPs in pre-service training. Some evidence suggests that therapist factors such as experience level may interact with EBP training to affect implementation outcomes, with some groups benefiting more than others (Bearman et al., 2013).

Therapist attitudes regarding evidence-based practice have been more consistently linked with observed EBP strategy delivery. Often assessed with the Evidence-Based Practice Attitudes Scale (EBPAS; Aarons, 2004), these attitudes include openness, or willingness to try new interventions and use EBPs, and divergence, or the attitude that research-based treatment manuals are not clinically useful and that clinical experience is more important than clinical research trials. Studies of CBT have found higher levels of therapist EBP strategy delivery associated with greater therapist openness to EBPs (Rodriguez, 2016) and lower levels of therapist divergence of EBPs (Ginsburg et al., 2019).

### The Current Study

In 2010, the Los Angeles County Department of Mental Health (LACDMH) launched the Prevention and Early Intervention (PEI) initiative which aimed to implement multiple EBPs for common youth mental health presenting problems. LACDMH furnished large-scale training for therapists in an initial set of six EBPs: Cognitive Behavioral Intervention for Trauma in Schools (CBITS), Youth-Parent Psychotherapy (CPP), Trauma-Focused Cognitive Behavior Therapy (TF-CBT), Triple P - Positive Parenting Program (Triple P), Seeking Safety (SS), and Managing and Adapting Practice (MAP).^1^ Within the context of this scale-up effort, the current study first sought to describe the patterns of therapist EBP strategy delivery using observational methods. Second, we examined associations between session-/EBP-, client-, and therapist-level factors and observed therapist EBP strategy use. Based on the EPIS framework, we examined potential predictors spanning across levels of the inner-context presumed to influence implementation outcomes (Aarons et al., 2011). These analyses were largely exploratory, given the paucity of existing literature in this area, however, we posed some directional hypotheses based on prior findings. We hypothesized that session/EBP-level factors such as the EBP requiring ongoing consultation and having a structured format would be associated with higher extensiveness of EBP strategy delivery. We also hypothesized that therapist factors including positive attitudes towards EBPs in general, and higher self-efficacy with a particular EBP would be associated with more extensive delivery of EBP strategies.

## Method

### Procedure

Data for the current study were collected as part of the Knowledge Exchange on Evidence-based Practice Sustainment (“4KEEPS”) study (Lau & Brookman-Frazee, 2016) and included 103 therapists working in 14 agencies. Therapists were eligible to participate in the current study if they were: (1) employed as a staff or trainee therapist in an agency contracted by LACDMH to deliver EBPs to youth, (2) trained in one of the six aforementioned EBPs of interest, and (3) reported currently delivering one of these EBPs to at least one client.

Research staff attended regular agency staff meetings to present information to therapists about study participation. Participation involved completion of an initial survey and semi-structured interview, as well as submission of audio recordings of sessions and questionnaires describing session characteristics. Each participating therapist could submit up to three sessions per client within a six-week period, for up to three clients. Audio recordings were made using a study-issued iPod Touch device. Therapists received a $20 incentive for completing the initial survey, a $10 incentive for each session recording, and $10 for completing the questionnaire associated with the session. Therapists were also permitted to keep the iPod used for recording following study completion after submitting a minimum number of sessions. Data were collected in 2015, approximately five years after the initial implementation effort. All procedures were approved by the Institutional Review Boards at [MASKED FOR REVIEW]. Therapist informed consent was obtained by research staff, and therapists obtained caregiver written permission and youth verbal assent for audio-recording of sessions.

### Participants

In the current study, data from 680 session audio recordings for 273 youth clients with 103 therapists from 14 agencies were examined. On average, each therapist submitted data for 2.65 clients (*SD* = 0.64), with an average of 2.49 sessions submitted per client (*SD* = 0.73). See Table 1 for additional details regarding session, client, and therapist characteristics.

**Table 1:** Descriptives of session, youth, and therapist characteristics

Characteristics	Session (*n* = 680)	Youth (*n* = 273)	Therapist (*n* = 103)
Session EBP, No. (%)			
CPP	49 (7.2)	–	–
CBITS	0 (0)	–	–
MAP	357 (52.5)	–	–
MAP – Anxiety	111 (16.3)	–	–
MAP – Conduct	153 (22.5)	–	–
MAP – Depression	83 (12.2)	–	–
MAP – Trauma	10 (1.5)	–	–
SS	27 (4.0)	–	–
TF-CBT	209 (30.7)	–	–
Triple P	38 (5.6)	–	–
Caregiver Present in Session, No. (%)	285 (41.9)	–	–
Language of Session, No. (%)			
English	564 (82.9)	–	–
Spanish	105 (15.4)	–	–
Mandarin	10 (1.5)	–	–
Cantonese	1 (0.2)	–	–
Therapist Self-Efficacy with EBP used in Session, M (SD)	3.9 (0.9)	–	–
Female Gender, No. (%)	–	138 (50.5)	91 (88.3)
Race/Ethnicity, No. (%)			
Non-Hispanic White	–	14 (5.1)	22 (21.4)
Hispanic	–	200 (73.3)	57 (55.3)
Black	–	44 (16.1)	9 (8.7)
Asian American/Pacific Islander	–	15 (5.5)	15 (14.6)
Age, M (SD); Range	–	9.8 (3.9); 1–18 years	–
Primary Diagnosis, No. (%)			
Anxiety	–	50 (18.3)	–
Attention or Hyperactivity Problems	–	7 (2.6)	–
Mood	–	55 (20.1)	–
Trauma	–	75 (27.5)	–
Disruptive Behavior or Conduct	–	79 (28.9)	–
Other	–	7 (2.6)	–
Education, No. (%)			
Less than Master’s Degree	–	–	4 (3.9)
Master’s Degree	–	–	88 (85.4)
Doctoral Degree	–	–	11 (10.7)
Theoretical Orientation, No. (%)			
Cognitive Behavioral	–	–	66 (64.1)
Humanistic	–	–	5 (4.9)
Family Systems	–	–	16 (15.5)
Psychodynamic	–	–	6 (5.8)
Eclectic/Other	–	–	10 (9.7)
Therapist Licensed, No. (%)	–	–	20 (19.4)
Years of Professional Experience, M (SD)	–	–	4.4 (4.4)
Number of EBPs Trained In, M (SD)	–	–	2.3 (0.9)
Therapist Emotional Exhaustion, M (SD)	–	–	2.3 (1.0)
Therapist General Attitudes towards EBPs, M (SD)			
Openness	–	–	3.0 (0.7)
Divergence	–	–	1.5 (0.8)

EBP = evidence-based practice; CPP = Youth-Parent Psychotherapy; CBITS = Cognitive Behavioral Intervention for Trauma in Schools; MAP = Managing and Adapting Practice; SS = Seeking Safety; TF-CBT = Trauma-Focused Cognitive Behavioral Therapy; Triple P = Positive Parenting Program. There were no sessions of CBITS included in this study, as this intervention was not widely implemented throughout the county.

**Table 2:** Standardized loadings (standard errors) for 2-factor confirmatory model of techniques

EBP strategies	Factor 1: Techniques related to structuring treatment		Factor 2: Techniques related to skill building
Establishing/Reviewing Agenda or Treatment Goals	0.86 (0.09)**		
Psychoeducation	0.84 (0.10)**		
Tracking/Reviewing Client’s Progress	0.78 (0.10)**		
Modeling			0.94 (0.06)**
Role Play & Practice			0.85 (0.08)**
Assigning/Reviewing Homework			0.60 (0.16)**
Delivering Positive Reinforcement & Rewards			0.90 (0.08)**

Results showed good model fit (RMSEA = 0.05; SRMR_within_ = 0.006, SRMR_between_ = -0.09; CFI = 0.96, TLI = 0.88; chi-square/df ratio = 2.96). * *p* < 0.05, ** *p* < 0.01.

### Measures

#### Outcome Variable: ECCA-α Observational Coding System (Brookman-Frazee et al., 2021)

*Content and Technique Items*. Audio recordings of therapy sessions were coded using the ECCA-α Observational Coding System (Brookman-Frazee et al., 2021), which includes items assessing therapist delivery of 32 EBP strategies considered essential for four common youth mental health problem targets – trauma, conduct, anxiety, and depression. Items assessed two types of strategies: (1) *content*, or the substance or issue addressed in the therapeutic intervention (24 items; e.g., time-out, exposure, trauma narrative, activity scheduling), and (2) *techniques*, the active method or the way a therapist attempted to intervene with, or relate to, a client (8 items; e.g., agenda setting, homework assignment and review, modeling, role play). The structure of the ECCA-α Observational Coding System was adapted from previous observational measurement systems, including the Practice and Research: Advancing Collaboration Therapy Process Observational Coding System for Youth Psychotherapy – Specific Therapy Process Scale (PRAC TPOCS-S; Garland et al., 2008a) and the Monthly Treatment and Progress Summary (MTPS; Child and Adolescent Mental Health Division, 2003).

*ECCA-α Observer Coding*. A total of 13 research staff (62% undergraduate, 38% post-baccalaureate) were trained in the ECCA-α Observational Coding System and coded audio recordings of each session. Each EBP strategy item was rated on a seven-point Likert scale for “extensiveness,” with a rating of 0 indicating that the EBP strategy was used “*not at all*,” a rating of 3 indicating that the EBP strategy was used “*to a moderate extent*,” and a rating of 6 indicating that the EBP strategy was used “*to a great extent*.” Coders were instructed to consider two related dimensions in rating the extensiveness of strategy use: (1) the thoroughness of strategy use (including effort, detail, depth/intensity, and follow-through), and (2) the frequency of strategy use (number of instances used during a session). Sessions delivered in Spanish were coded by Spanish speaking trained coders. For sessions in Mandarin and Cantonese, a native speaker interpreter provided real-time interpretation as a trained coder listened to the recording and coded the session.

Coder training included manual review, didactic training sessions, and practice coding. Coders were considered reliable and ready to begin independent coding when they achieved at least 80% agreement for each of the six or more criterion rated recordings. Coders were randomly assigned to each session and were kept naïve to the EBP being delivered and the problem target addressed. 26% of the sessions were randomly selected for double coding for purposes of evaluating inter-rater reliability. Observer inter-rater reliability was calculated using a one-way random effects *ICC*_(1,*k*)_ model based on a mean-rating, absolute-agreement (Hallgren, 2012; McGraw & Wong, 1996). Using the standards outlined by Cicchetti (1994), inter-rater reliability between observer coders for the 32 items was acceptable (all *ICC*’s of at least 0.40), and 30 of the items had inter-rater reliability estimates in the good to excellent range (*ICC*’s of at least 0.60). The average *ICC*_(1,*k*)_ for all items was 0.74 (*SD* = 0.11; range from 0.44 (Monitoring) to 0.92 (Trauma Narrative).

*ECCA-α Observer Composites*. Three composites were calculated from the coding: one content composite and two technique composites.

*ECCA-α Observer Content Composite.* For content items, the *ECCA Top Content Composite* was calculated by averaging the top two highest rated content items in each session. This scoring method was based on similar rating methods and on the theory that individual content strategies are not expected to be present in every session (Kolko et al., 2011; Morgan et al., 2018). Indexing the extensiveness of the two content strategies delivered in a session gauged the depth with which a therapist delivered focal therapeutic interventions.

*ECCA-α Observer Technique Composites.*For the technique items, a two-level confirmatory factor analysis (Level 1: sessions, Level 2: therapist) was used to evaluate a two factor model based on prior work (Hurwich-Reiss et al., 2021; see Table 2). The two factors were: (1) *ECCA Structuring Treatment Techniques* (this included the strategies: Establishing/Reviewing Agenda, Psychoeducation, and Tracking/Reviewing Client’s Progress), and (2) *ECCA Skill Building Techniques* (this included the strategies: Modeling, Role Play & Practice, Assigning/Reviewing Homework, and Delivering Positive Reinforcement & Rewards). Maximum likelihood parameter estimates (MLR) were used to estimate model parameters and goodness-of-fit of the CFA model. Results showed good model fit (RMSEA = 0.05; SRMR_within_ = 0.006, SRMR_between_ = -0.09; CFI = 0.96, TLI = 0.88; chi-square/df ratio = 2.96). See Table 2 for the standardized loadings. Based on these results, two technique composites were computed: (1) *ECCA Structuring Treatment Techniques*, and (2) *ECCA Skill Building Techniques*. These two factors were significantly correlated (*r* = 0.37, *p* < 0.01).

**Table 3:** Session/EBP-, youth-, and therapist-level predictors of observed therapist EBP strategy delivery

	Content		Techniques			
	ECCA Top Content		ECCA Structuring Treatment Techniques		ECCA Skill Building Techniques	
	*B (SE)*	95% CI	*B (SE)*	95% CI	*B (SE)*	95% CI
*Session/EBP Variables*						
Caregiver Involved in Session	− 0.02 (0.14)	− 0.29, 0.25	0.20 (0.10)	− 0.001, 0.41	− 0.07 (0.11)	− 0.28, 0.15
Session Conducted in Non-English Language	− 0.04 (0.18)	− 0.39, 0.31	− 0.06 (0.14)	− 0.34, 0.22	0.36 (0.15)*	0.06, 0.65
Session EBP Structured Content	0.92 (0.18)**	0.58, 1.27	0.27 (0.15)	− 0.03, 0.57	0.29 (0.15)	− 0.02, 0.59
Session EBP Ongoing Consultation	− 0.47 (0.25)	− 0.95, 0.01	− 0.35 (0.22)	− 0.77, 0.08	− 0.39 (0.21)	− 0.81, 0.03
Therapist Self-Efficacy for Specific Session EBP	0.31 (0.09)**	0.12, 0.49	0.26 (0.08)**	0.10, 0.43	0.04 (0.08)	− 0.11, 0.20
Client Variables						
Client Gender (Ref = Female)	− 0.02 (0.12)	− 0.25, 0.21	− 0.05 (0.10)	− 0.25, 0.15	− 0.02 (0.11)	− 0.24, 0.19
Client Age	− 0.01 (0.02)	− 0.04, 0.03	0.03 (0.02)*	0.003, 0.06	− 0.04 (0.02)*	− 0.07, − 0.01
Therapist Variables						
Number of EBPs Trained In	− 0.10 (0.09)	− 0.28, 0.07	0.07 (0.08)	− 0.08, 0.23	0.07 (0.07)	− 0.07, 0.21
Therapist Emotional Exhaustion	− 0.07 (0.08)	− 0.09, 0.22	0.10 (0.07)	− 0.04, 0.25	− 0.04 (0.07)	− 0.17, 0.09
Therapist General Attitudes Towards EBP – Divergence	0.07 (0.09)	− 0.12, 0.25	− 0.16 (0.09)	− 0.33, 0.01	− 0.09 (0.08)	− 0.25, 0.06
Therapist Cognitive Behavioral/Behavioral Orientation	0.27 (0.16)	− 0.05, 0.59	0.31 (0.15)*	0.01, 0.60	0.19 (0.14)	− 0.07, 0.46

Target of session (i.e., trauma, conduct, anxiety, or depression) was controlled for in all analyses. The following variables were found to have no significant association with observed therapist EBP strategy delivery in the univariate models and therefore were not included in the full models: youth race/ethnicity, youth-therapist race/ethnicity match, therapist gender, therapist race/ethnicity, therapist licensure status, therapist number of years practiced, and general openness towards EBPs. * *p* < 0.05, ** *p* < 0.01.

#### Predictor Variables: Session/EBP-Level Factors

*Session Language, Participants, & EBP Focus*. In the questionnaire completed after the session, therapists identified the language of the session (83% English, 15% Spanish, 2% Mandarin or Cantonese) and whether a caregiver was involved (42% of sessions had a caregiver present). Therapists also indicated which of six EBPs was delivered in the session – CBITS, CPP, MAP (specifying MAP Anxiety, MAP Conduct, MAP Depression, MAP Trauma), Triple P, TF-CBT, or SS. Practices were grouped based on their primary mental health treatment target into: Trauma (CBITS, CPP, MAP Trauma, TF-CBT, Seeking Safety), Conduct (Triple P, MAP Conduct), Anxiety (MAP Anxiety), and Depression (MAP Depression). See Table 1 for descriptive information.

*EBP Structure: Prescribed Session Content/Order*. EBPs were characterized based on their structure, with EBPs with prescribed session content and order defined as those with explicit guidance in their manuals as to what content should be covered in which order. Based on guidance in their manuals, three of the EBPs, CBITS (Jaycox, 2003), TF-CBT (Cohen et al., 2017), and Triple P (Turner et al., 2002) were classified as having this structure. In contrast, Seeking Safety (Najavits, 2001), CPP (Lieberman et al., 2015), and MAP (Chorpita et al., 2014) were classified as having more flexibility in the selection and ordering of therapy content and activities across an episode of treatment. Following Barnett et al., (2017), EBPs were classified as having a prescribed session content and order (CBITS, Triple P, and TF-CBT) were coded as a 1, and the practices without a prescribed session content/order (Seeking Safety, CPP, and MAP) were coded as a 0.

*EBP Implementation Requirements: Consultation*. EBPs were also characterized on whether or not there was mandatory follow-up consultation with an EBP expert trainer after the initial training. These implementation requirements were established for the PEI implementation initiative by LACDMH in consultation with the EBP developers (Los Angeles County Department of Mental Health, 2016). Seeking Safety and Triple P did not require ongoing consultation for therapists delivering the practice for PEI reimbursement, whereas CBITS, CPP, MAP, and TF-CBT did, although the length and format of the required consultation varied. Following Barnett et al. (2017), the EBPs classified as having required consultation were coded as a 1, and those without required consultation were coded as a 0.

*Therapist EBP Self-Efficacy*. Therapist self-efficacy, or their belief in their capacity to deliver a practice, was assessed with two items. Therapists reported on feelings of self-efficacy for each of the EBPs they had delivered: “I am well prepared to deliver [EBP] even with challenging clients,” and “I am confident in my ability to implement [EBP].” Therapists responded on a 5-point Likert scale (1 = not at all, 5 = a very great extent). A mean of both items was used as a composite to indicate therapists’ feelings of efficacy with the EBP being delivered in that particular session (α = 0.83 to 0.94 across EBPs).

#### Predictor Variables: Client-Level Factors

*Client Characteristics*. Therapists reported basic client demographic information including age, gender, and race/ethnicity of the youth client. Youth and therapist race/ethnicity were reported as: Non-Hispanic White, Hispanic, Black, Asian American/Pacific Islander, American Indian, and Multiracial. Based on the characteristics of the sample, these were re-coded into four categories: Non-Hispanic White, Hispanic, Black, and Asian American/Pacific Islander. A therapist-youth race/ethnicity match was coded if the youth and therapist were reported as having the same race/ethnicity. The majority (61.9%) of clients had a race/ethnicity match with their therapist.

#### Predictor Variables: Therapist-Level Factors

*Therapist Demographic, Professional, and Practice Characteristics*. In the initial survey, therapists reported information concerning personal and professional characteristics, including licensure status, number of years practiced, number of EBPs trained in, and theoretical orientation. See Table 1 for additional details.

*Therapist Emotional Exhaustion*. Therapists responded to 5 items from the Emotional Exhaustion subscale of the Organizational Social Context Questionnaire (OSC; Glisson et al., 2008). Therapists rated their perceptions of stressful climates characterized by factors such as workload (e.g., “I feel used up at the end of the day”) and work-related emotional exhaustion (e.g., “I feel emotionally drained from my work”). Responses were on 7-point Likert Scale (0 = strongly disagree, 6 = strongly agree). A mean score was calculated, with higher scores representing more emotional exhaustion. The current sample demonstrated good internal consistency of *α =* 0.81 using Cronbach’s alpha.

*Therapist General Attitudes Towards EBPs*. Therapists’ general attitudes towards the adoption of EBPs was measured with the Evidence-Based Practice Attitudes Scale (EBPAS; Aarons, 2004). The current study included two subscales: openness and divergence, each of which consisted of four items. The Openness subscale assessed the therapist’s openness to trying new interventions and willingness to use EBPs (e.g., “I like to use new types of therapy/interventions to help my clients”). The Divergence subscale assessed the perception that EBPs were not useful or were not aligned with the therapist’s approach to practice (e.g., “Research based treatments/interventions are not clinically useful”). Therapists rated each item on a five-point Likert scale (0 = not at all, 4 = very great extent) and a mean score was calculated for each subscale. In the current sample, internal consistency was acceptable for Openness (*α* = 0.79) and Divergence (*α* = 0.71).

### Data Analytic Plan

Aim 1 sought to describe the patterns of observed therapist EBP strategy use. Aim 2 sought to examine possible session-/EBP-, client-, and therapist-level predictors of observed therapist EBP strategy use as measured by the *ECCA Top Content Composite*,* ECCA Structuring Treatment Techniques Composite*, *ECCA Skill Building Techniques Composite.* An unconditional model indicated significant variance in these three composites attributable to the client, therapist, and agency levels. A significant proportion of variance was attributable to the client level (*ICC*s = 0.30–0.47) and the therapist level (*ICC*s = 0.21–0.30), and a small proportion of variance was attributed to the agency level (*ICC*s = 0.01–0.03). To account for the nested structure of the data, analyses employed a four-level model with session observations (Level 1; *n* = 680), nested within clients (Level 2; *n* = 273), nested within therapists (Level 3; *n* = 103), nested within agencies (Level 4; *n* = 14).

Exploratory univariate, multilevel models were run to examine potential associations of session/EBP-, client-, and therapist-level factors with each of the three composites. Predictors included session/EBP variables (caregiver present in session, session conducted in a non-English language, whether EBP delivered in session had a prescribed content/order, whether EBP delivered in session had required consultation, and therapist reported self-efficacy with EBP delivered in the session), client variables (youth age, gender, race/ethnicity, youth-therapist race/ethnicity match), and therapist variables (therapist gender, race/ethnicity, licensure status, number of years practiced, number of EBPs trained in, theoretical orientation, emotional exhaustion, general attitudes towards EBPs, and perceptions of agency organizational climate). Variables that were associated with observed therapist EBP strategy use at *p* < 0.10 were retained for the full models. Three separate four-level multilevel models were run to examine the associations between session/EBP, youth, and therapist variables and the following composites: (1) *ECCA Top Content*, (2) *ECCA Structuring Treatment Techniques*, and (3) *ECCA Skill Building Techniques*. All analyses were run using Stata SE 7.

## Results

### Aim 1: Describe Patterns of Observed Therapist EBP Strategy Use

#### Content Strategies

**Fig. 1:**
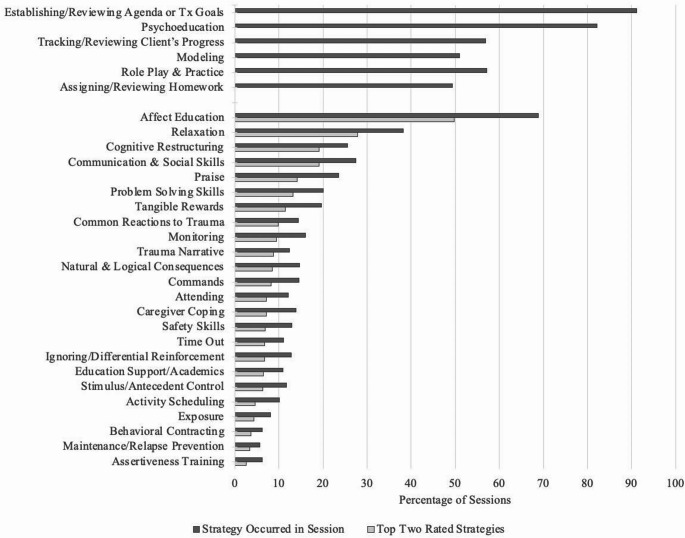
Percentage of sessions with strategy present

Figure 1 shows how often each content strategy was among the highest or lowest rated in a session. The content items occurred most frequently in sessions were: Affect Education (49.7% of sessions), Relaxation (27.9% of sessions), and Cognitive Restructuring (19.2% of sessions), and items that occurred the least frequently were Assertiveness Training (2.6% of sessions) and Maintenance/Relapse Prevention (3.4% of sessions).

#### Content Composite

**Fig. 2:**
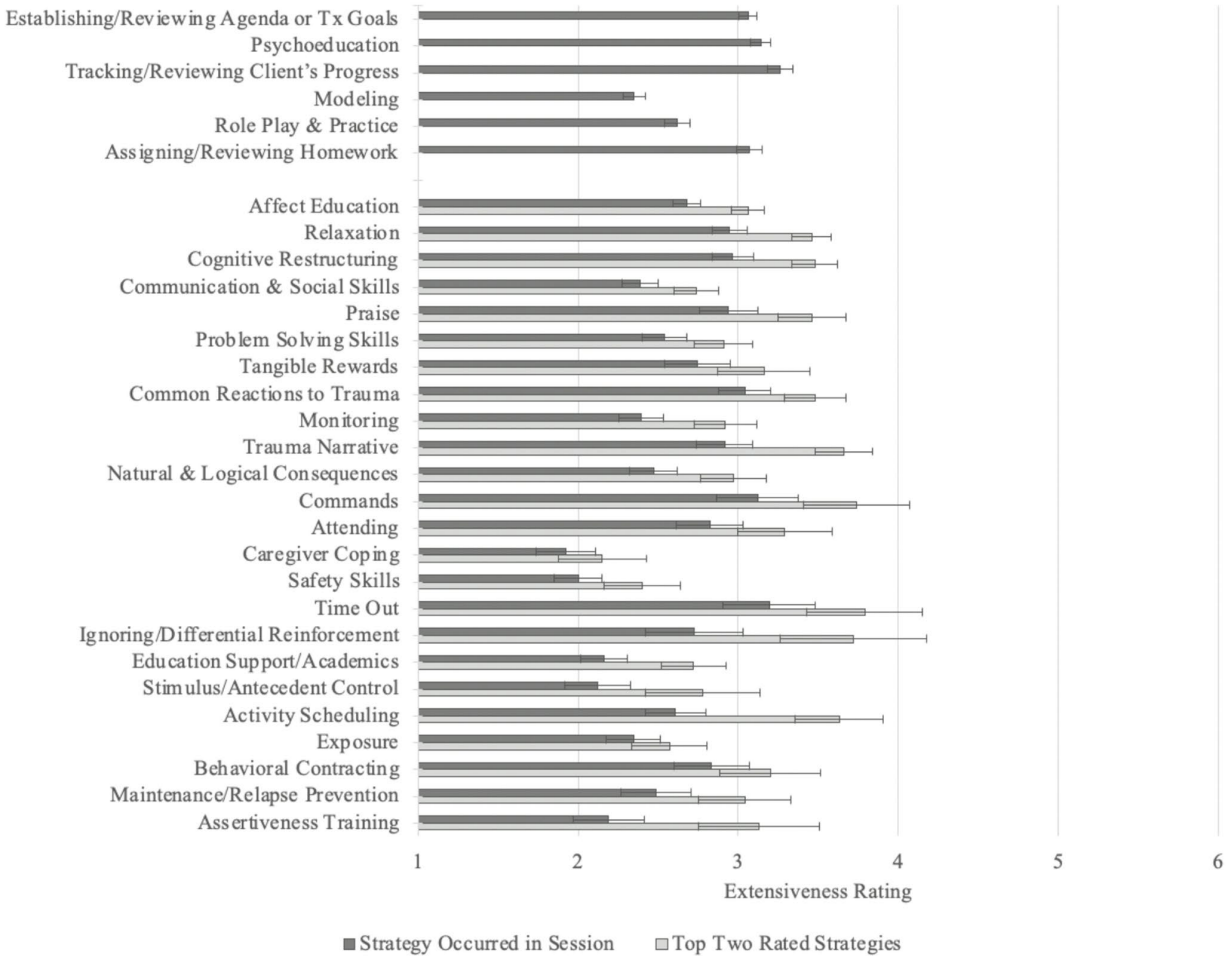
Extensiveness ratings of strategies present

Scores on the *ECCA Top Content Composite* ranged from 0 to 6 (*M* = 2.93, *SD* = 1.48). Figure 2 shows the extensiveness ratings for each content strategy rated in the top two. Figure 2 shows that the top two content items were rated to occur “to a great extent” 40% of the time, “to a moderate extent” 36% of the time, “to a minimal extent” 19% of the time, and “not at all” 5% of the time.

#### Technique Strategies

Figure 1 shows percentage of sessions that contained each technique strategy. The technique strategies that occurred the most frequently were: Establishing/Reviewing Agenda or Treatment Goals (91.2% of sessions) and Psychoeducation (82.1% of sessions). The least commonly occurring techniques were: Modeling (51.0% of sessions) and Assigning/Reviewing Homework (49.4% of sessions). Figure 2 shows the extensiveness ratings for each technique strategy. The strategies of Role Play/Practice (*M* = 3.26, *SD* = 1.56) and Psychoeducation (*M* = 3.14, *SD* = 1.52) had the highest extensiveness ratings when they occurred.

#### Technique Composites

Scores on the *ECCA Structuring Treatment Techniques* composite ranged from 0 to 5.33 (*M* = 2.24, *SD* = 1.17), and scores on the *ECCA Skill Building Techniques* composite ranged from 0 to 5.75 (*M* = 1.75, *SD* = 1.19).

### Aim 2: Examine Session-/EBP-, Client-, and Therapist-Level Predictors of Observed Therapist EBP Strategy Use

Exploratory univariate, multilevel models were run to examine potential associations of session/EBP-, client-, and therapist-level factors with each of these three composites. Predictors included session/EBP variables (caregiver present in session, session conducted in a non-English language, whether EBP delivered in session had a prescribed content/order, whether EBP delivered in session had required consultation, and therapist reported self-efficacy with EBP delivered in the session), client variables (youth age, gender, race/ethnicity, youth-therapist race/ethnicity match), and therapist variables (therapist gender, race/ethnicity, licensure status, number of years practiced, number of EBPs trained in, theoretical orientation, emotional exhaustion, general attitudes towards EBPs, and perceptions of agency organizational climate).

The following variables were found to have no significant association with observed therapist EBP strategy delivery: youth race/ethnicity, youth-therapist race/ethnicity match, therapist gender, therapist race/ethnicity, therapist licensure status, therapist number of years practiced, and general openness towards EBPs. Variables that were associated with observed therapist EBP strategy use at *p* < 0.10 were retained for the full models (this included: caregiver involvement in session, session language, session had EBP with structured content, session had EBP with ongoing consultation, client gender, client age, therapist self-efficacy with EBP, number of EBPs therapist trained in, therapist emotional exhaustion, therapist general attitudes towards EBPs – divergence, and therapist cognitive-behavioral orientation). Three separate four-level multilevel models were run to examine the associations between these session/EBP, client, and therapist variables and the following composites: (1) *ECCA Top Content*, (2) *ECCA Structuring Treatment Techniques*, and (3) *ECCA Skill Building Techniques*.

#### Associations with ECCA Top Two Content Composite

Results of the multilevel models showed that sessions with an EBP with a structured order or content had significantly more EBP content strategy delivery as measured by the *ECCA Top Content* composite than sessions without an EBP with a structured order or content (*B* = 0.92, *p* < 0.01). Other results showed that therapists who reported higher levels of self-efficacy for the specific EBP delivered in session had higher levels of observed content delivery (*B* = 0.31, *p* < 0.01).

#### Associations with ECCA Technique Composites

For *ECCA Structuring Treatment Techniques*, therapists who reported having a cognitive behavioral or behavioral orientation were observed to use more structuring techniques, compared with therapists who reported other types of theoretical orientation (*B* = 0.31, *p* < 0.05). In addition, therapists who reported higher levels of self-efficacy for the specific EBP delivered in the session had higher levels of strategy delivery on this composite (*B* = 0.26, *p* < 0.01). In terms of client characteristics, older youth age was associated with higher levels of structuring techniques (*B* = 0.03, *p* < 0.05).

For *ECCA Skill Building Techniques*, sessions conducted in a non-English language had significantly higher use of skill building techniques, compared with sessions delivered in English (*B* = 0.36, *p* < 0.05). Older youth client age was associated with lower levels of skill building techniques (*B* = -0.04, *p* < 0.05).

## Discussion

The goals of the present study were to: (1) describe observed delivery of EBP strategies for children and youth in community-based services several years into a large-scale, multi-EBP implementation effort, and (2) identify session-, client-, and therapist-level predictors of observed extensiveness of EBP content and technique strategies. Descriptive analyses revealed content and technique strategies that were observed the most frequently (Affect Education, Establishing/Reviewing Session Agenda) and least frequently (Assertiveness Training, Assigning/Reviewing Homework) across sessions. With regard to extensiveness of strategy delivery, the majority of clinicians delivered content strategies with relatively high extensiveness, such that the top two content strategies by problem target were delivered to a “great” or “moderate” extent in 76% of sessions. Similarly, 87% of sessions were observed to include structuring treatment or skill building techniques to a “great” or “moderate” extent. Finally, a number of session/EBP, client, and therapist variables were found to predict observed EBP content and/or technique strategies, including the language primarily spoken during the session, structural qualities of the EBP being delivered, therapist self-efficacy, client age, and therapist theoretical orientation. Given the dearth of observational measurement capturing EBP service delivery in community mental health settings at large, these findings contribute to our understanding of how EBP strategies are delivered in this context and provide some insight about how to support EBP implementation in community settings.

First, we discuss our descriptive findings within the context of other studies employing detailed observational measurement. Although it not possible to statistically benchmark our ratings with those observed in prior studies with similar measures, we found that, in general, extensiveness scores observed in this study are on par with similarly ascertained ratings from other EBP delivery contexts. For example, observers using the Cognitive-Behavioral Therapy Adherence Scale for Youth Anxiety employed a similar seven-point extensiveness scale comparable to the content scores described in the current study to rate therapist delivery of individual CBT in both an effectiveness and efficacy trial (Southam-Gerow et al., 2010). Results shows that community-based clinicians delivering individual CBT as a part of an effectiveness trial were rated by observers to be approaching “moderate” extensiveness, and research-setting-based clinicians in an efficacy trial at “high-moderate” extensiveness (Southam-Gerow et al., 2010, 2016). In the current study, the average content score falling in the “moderate” extensiveness range appeared to be on par with the ratings found in the effectiveness trial (Southam-Gerow et al., 2010, 2016).

### EBP-Level Factors Associated with Observed Therapist EBP Strategy Delivery

Next, we consider how the session/EBP, client, and therapist variables that emerged as significant predictors in the multilevel analyses and how they can be understood within the larger literature. Among session/EBP-level variables examined, therapist self-efficacy with the specific EBP being delivered in the session was found to predict higher observed extensiveness of content strategy delivery and techniques related to structuring. This may be among the most actionable findings of this study, considering the theorized relation between therapist self-efficacy, EBP training, and implementation outcomes. Broadly, self-efficacy has a robust relation to task performance (Bandura, 1977) and it has emerged as a variable of interest to the process of implementing EBPs for youth across the breadth of the mental health workforce (Gallo & Barlow, 2012). The Longitudinal Education for Advancing Practice (LEAP) model, which aims to identify training inputs, training and consultation strategies, and mechanisms of learning that influence clinical training outcomes, highlights self-efficacy as a core affective mechanism that may drive clinical training (McLeod et al. 2018a, b). Empirical investigations of the relationship between therapist self-efficacy and observed therapist behavior is less well-explored. The only available example is a study conducted with therapists learning Cognitive Processing Therapy, which found no significant association between therapist self-efficacy and therapist behavior in sessions as rated by observers (Pace et al., 2021). In contrast, findings from the present study suggest that session-level self-efficacy did predict observer-rated extensiveness of both EBP content and technique strategies. In essence, this study provides evidence of the validity of community therapists’ self-reports of EBP self-efficacy.

Given this association, several strategies may be targets to increase therapist self-efficacy in implementation efforts. For example, clinicians who received regular clinical supervision, consultation and performance feedback have been shown to report a higher level of self-efficacy in their general counseling skills (Cashwell & Dooley, 2001; Daniels & Larson, 2001; Gibson et al., 2009; Mehr et al., 2015). Studies of specific EBP implementation, such as Cognitive Processing Therapy (CPT), have also shown that ongoing supervision and consultation is associated with higher levels of therapist self-efficacy with the EBP (Pace et al., 2021). More recent work has highlighted the role that AI-assisted and online counseling training can have in improving therapists’ general self-efficacy (Jeong et al., 2025; Vincenzes et al., 2023). In sum, providing ongoing supervision and consultation may be a critical actionable area in increasing therapist self-efficacy, and therefore EBP strategy delivery, in implementation efforts.

Other significant session/EBP variables associated with therapist EBP delivery were EBP structure and language used in session. Sessions involving EBPs with structured session content and order had higher observed extensiveness of EBP content delivery than those sessions where a less structured EBP was being delivered. This result is unsurprising given the manualized nature of these structured EBPs, but provides support for the use of explicit guided resources to increase EBP content delivered in session. Sessions conducted in a non-English language were associated with higher use of skill building techniques. This finding may be testament to the responsiveness of community therapists’ addressing barriers to learning and using more extensive active teaching strategies skills for non-English speaking families in settings serving diverse youth and families in Los Angeles County. Such responsiveness may indicate that bilingual therapists are particularly well equipped to transcend barriers to engagement that have been found in prior studies showing lower average treatment involvement among caregivers of racially/ethnically minoritized youth (Barnett et al., 2020). For example, in addition to verbal didactics and psychoeducation, it appears that therapists working with non-English speaking clients employ more in-session demonstration and rehearsal. Prior findings in the current study context suggest that Latinx therapists also report making more augmenting cultural adaptations to EBPs and report fewer barriers to engagement than non-Hispanic White therapists (Lau et al., 2017, 2018; Ramos et al., 2021). It is also possible that Spanish-speaking clients tend to exhibit higher levels of skill building strategies (e.g., positive reinforcement), and that therapists therefore tend to call more attention to these strategies in Spanish language sessions. Of note, therapist ethnicity was not associated with EBP strategy delivery. In the context of this study, a large proportion of non-Hispanic therapists can deliver services in Spanish. This may help to explain the differing findings between therapist ethnicity and language. Overall, these findings together reinforce the critical importance of diversifying the community mental health workforce and actively recruiting more bilingual bicultural therapists who are in an excellent position to providing culturally responsive and high-fidelity delivery of EBPs to diverse youth and families.

It is also important to consider what session/EBP-level variables were unrelated to extensiveness of EBP strategy delivery. Delivering an EBP that required ongoing consultation was not significantly associated with observed therapist EBP content and technique delivery relative to delivering EBPs that had no consultation requirement. This result adds to the mixed findings concerning the benefits of consultation on observed therapist EBP strategy delivery (Caron & Dozier, 2019; Wain et al., 2015). Given that we did not directly measure the form, duration, and actual attendance of therapists in ongoing consultation across EBPs and where in the consultation process therapists were, it is possible that the effect of consultation could not be discerned in this multi-EBP implementation context. Proximal measures of activities that occur in ongoing EBP consultation are required to identify what support is necessary for improving therapist strategy delivery. A recent study examining supervision of therapists in community EBP implementation context found that discussion of EBP strategies occupied only 20.2% of overall time in supervision, and supervisor EBP competence was rated as low by trained observers (Bailin & Bearman, 2021). If community therapists in the present study had similar experiences in consultation, this may further explain why we did not observe associations with therapist strategy delivery. It remains important to identify specific consultation practices that may promote sustained EBP use and fidelity.

### Client-Level Factors Associated with Observed Therapist EBP Strategy Delivery

Among client characteristics, only age was associated with observed EBP strategy delivery, with older age being associated with higher use of structuring techniques and younger age with use of more extensive skill building techniques. Similar to working with non-English speaking families, therapists may rely on active teaching skills (e.g., modeling, positive reinforcement) rather than didactic structuring techniques (e.g., psychoeducation) with younger clients and their caregivers. While the extant literature on the relationship of client age and EBP strategy delivery is mixed, this finding is consistent with suggestions to developmentally tailor EBP strategy delivery for younger children in Pivotal Response Treatment (Suhrheinrich et al., 2016) and CBT for anxiety (Kendall & Peterman, 2015). Client-level variables that were unrelated to observed strategy delivery were client gender, race/ethnicity, and therapist-client race/ethnicity. Although the finding of therapist-client match is contrary to that of previous studies of MST, it is possible that the diverse sample of clients and providers from Los Angeles County make this match less salient to treatment adherence than it was for clients and therapists in the MST studies, who primarily identified as White (Chapman & Schoenwald, 2011).

### Therapist-Level Factors Associated with Observed Therapist EBP Strategy Delivery

For therapist-level variables, only therapist orientation, specifically those who identified with a cognitive-behavioral or behavioral orientation, was associated with higher observed use of EBP structuring techniques. This is consistent with principles of CBT, which is present-focused, active, skill-based, as well as common components of CBT sessions, such as agenda setting, teaching skills assessing and measuring progress (Gosch et al., 2006). There was no significant association between therapist licensure, years of experience, and observed EBP content and technique delivery. Although the literature on the impact of therapist-level factors on observed EBP use is mixed as described earlier (e.g., Beidas et al., 2014; Ginsburg et al., 2019), these findings suggest that the emerging workforce in this context is able to deliver EBP strategies at the same intensity level as more experienced therapists.

### Limitations & Future Directions

There are a number of limitations to consider. First, observational data were available only at the session level, so there was no information regarding treatment phase or other dimensions of the therapy process. These data may be helpful in better understanding determinants of core EBP strategies that are typically deployed in established sequence (e.g., trauma narrative being a focus later in a therapy episode). Additionally, it would help expand upon whether certain EBP content occurred more often than other content in sessions based on where in the therapy process clients were. For example, affect education and relaxation may have occurred in more sessions because they are strategies typically used in the beginning of the therapy sequence and there is more drop-out from therapy at later sessions (Dorsey et al., 2014) The ECCA-α extensiveness rating scale itself also has limitations. Though observational ratings were linked to explicit coding anchors, it is difficult to quantify how meaningful the differences between “low,” “moderate,” and “high” extensiveness are. There is also a concern of self-selecting bias in the therapists who chose to participate in this study. Only a portion of therapists who received the EBP training were included in the study. Therapists who used more EBP strategies may have been more inclined to participate. In addition, therapists may have be inclined to nominate cases/sessions to submit to the study they feel went “well,” where they felt they were delivering more EBP strategies. Therapists were asked to choose cases at random, but this selection bias could influence the results. Finally, there is some circularity inherent to the finding that EBPs with structured content were associated with higher use of EBP content strategies, given the ECCA-α’s emphasis on cognitive and behavioral strategies. In refining observer ratings of EBP delivery, it will be critical to think about how best to characterize what therapists are doing when delivering more process- and/or principle-based EBPs.

These limitations notwithstanding, the present study contributes to our understanding of community therapist EBP strategy delivery through observational measurement of EBP implementation-as-usual. Findings characterize the EBP delivery patterns in this service context, in which technique strategies were observed in the majority of sessions and some specific content strategies, namely affect education and relaxation were used frequently. In addition, predictors of EBP strategy use were identified at the session/EBP-, client-, and therapist-level, suggesting various targets for tailored implementation strategies to improve EBP delivery and sustainment.

## Data Availability

No datasets were generated or analysed during the current study.

## Footnotes

1 We use the term “EBPs” throughout, inclusive of MAP, for ease of communication. We acknowledge that MAP differs from the other EBPs as it is an evidence management system, with a direct service component that coordinates multiple evidence sources, employs practices components drawn from over 700 evidence-based treatments, and offers structured process management supports to guide clinical care for anxiety, trauma, depression, conduct problems in children 0–21 years old. In the LACDMH system, the MAP direct service model complements a diverse array of EBPs by allowing therapists to select, review, adapt, or construct promising treatments as needed to match particular youth characteristics based on the latest scientific findings (see Chorpita & Daleiden, 2018).
